# Assessment of the trabeculated to compacted layer ratio of the left ventricle using short and longitudinal axis views in young patients presenting with ventricular dysrhythmias

**DOI:** 10.1186/1532-429X-15-S1-P62

**Published:** 2013-01-30

**Authors:** Moneal Shah, Lynette Duncanson, Ellen Cummings, Jie J Cao

**Affiliations:** 1 Allegheny General Hospital, Pittsburgh, PA, USA; 2 Cardiology, St. Francis Hospital, Rosyln, NY, USA

## Background

Cardiac MRI (CMR) is increasingly being used clinically to assess the regional thickness of the compacted and trabeculated layers of the left ventricle (LV) in patients with suspected non-compaction. However, there is no consensus on whether the short or longitudinal axis view is preferred. We sought to assess the compacted and trabeculated layers in a young ventricular dysrhythmia population with age matched controls and to compare the measurements derived from both the short axis and longitudinal views.

## Methods

The study consisted of 91 patients aged 20 to 49 years with ventricular dysrhythmia (VT) and 23 age-matched normal subjects. Regional wall thickness was assessed in short axis CMR cine views using a basal, mid and apical slice as well as in longitudinal views using the 2, 3, and 4 chamber view. The images were analyzed at end-diastole where the compacted layer was measured following the standardized 16-segment model. The apical segments were chosen 2 cm from the most apical portion of the LV for both short and longitudinal views. Within each wall segment, the most prominent trabeculation was measured, and the ratio of the trabeculated to compacted layer was compared between the VT group and controls in both views.

## Results

The mean age was 38 years and 48 (42%) were men. In the VT group, 31 had premature ventricular contractions and 60 had ventricular tachycardia. VT patients were associated with larger LV end-diastolic volumes (89.7 ml/m2 vs. 82.6 ml/m2, p=0.05), larger LV mass (58 g/m2 vs. 49.9 g/m2 p=0.001), and lower LV ejection fraction (50% vs. 60% p=0.015) as compared to the control group, respectively. Using the short axis view, the prevalence of trabeculated to compacted layer ratios ≥ 2.0, ≥ 2.1, ≥ 2.2 and ≥ 2.3 were 18.4%, 15.2%, 9.2% and 8.7% in the VT group, respectively while the prevalence in the control group was 13% for a ratio ≥ 2.0 with no segments ≥ 2.1. Using the same ratio cut points for the longitudinal view, the prevalence was 39.1%, 32.6%, 27.2% and 16.3% in the VT group and 43%, 39.1%, 21.7%, and 13% in controls, respectively.

## Conclusions

In this young cohort, the trabeculated to compacted ratio ≥ 2.1 is only present in patients with ventricular dysrhythmias and not seen in normal controls using short axis planes, suggesting that increased thickness of the trabeculated layer in young subjects may be associated with the risk of ventricular dysrhythmia. However, the ratio ≥ 2.3 is equally prevalent in both groups using longitudinal planes. Our findings suggest that the short axis view may be more discriminatory then the longitudinal view in identifying normal subjects. More importantly, there is a clear need to standardize the assessment of the trabeculated to compacted layer ratio in clinical practice.

## Funding

None

